# Effect of *Tithonia diversifolia* (Hemsl.) A. Gray intake on in vivo methane (CH_4_) emission and milk production in dual-purpose cows in the Colombian Amazonian piedmont

**DOI:** 10.1093/tas/txac139

**Published:** 2022-12-03

**Authors:** Julián Esteban Rivera, Gonzalo Villegas, Julian Chará, Sandra G Durango, Miguel A Romero, Louis Verchot

**Affiliations:** Centro Para la Investigación en Sistemas Sostenibles de Producción Agropecuaria, CIPAV, Cali, Valle de Cauca, 760002, Colombia; Centro Para la Investigación en Sistemas Sostenibles de Producción Agropecuaria, CIPAV, Cali, Valle de Cauca, 760002, Colombia; Centro Para la Investigación en Sistemas Sostenibles de Producción Agropecuaria, CIPAV, Cali, Valle de Cauca, 760002, Colombia; Alliance Bioversity International, International Center for Tropical Agriculture, Km 17 recta Cali-Palmira, Cali, Valle de Cauca, 763537, Colombia; Alliance Bioversity International, International Center for Tropical Agriculture, Km 17 recta Cali-Palmira, Cali, Valle de Cauca, 763537, Colombia; Alliance Bioversity International, International Center for Tropical Agriculture, Km 17 recta Cali-Palmira, Cali, Valle de Cauca, 763537, Colombia

**Keywords:** enteric fermentation, forage shrub, GHG mitigation, grazing, milk yield, silvopastoral systems

## Abstract

The inclusion of *Tithonia diversifolia* in pasture-based diets is a promising alternative to increase bovine productivity, due to its chemical composition and wide adaptation, but there are few in vivo studies to determine its effect on methane yield and animal production in grazing systems. The objective of this study was to determine the effects of the *T. diversifolia* inclusion in a basal diet of *Brachiaria humidicola* on methane (CH_4_) emissions by enteric fermentation, and on milk yield and quality in dual-purpose cows. The polytunnel technique was used for the determination of methane yield and two diets were evaluated (Diet 1: *Brachiaria humidicola* 100%; Diet 2: *T. diversifolia* 15% + *B. humidicola* 85% dry matter basis) in the moderate rainy and rainy seasons using a *cross-over* experimental design; milk production was measured by daily milk weighing, and milk quality was determined using a LACTOSCAN analyzer. The inclusion of *T. diversifolia* did not increase the dry matter intake (*P *= 0.369), but increased the intake of crude protein and minerals, and reduced fiber intake, resulting in the increased yield of milk and its components in the moderate rainy season (*P *= 0.012). The inclusion of *T. diversifolia* reduced the absolute CH_4_ emissions (*P *= 0.016), Ym and emission intensity (per unit of fat, protein and kilogram fat and protein corrected milk yields) both in the moderate rainy and rainy seasons (*P* < 0.05). We conclude that the inclusion of *T. diversifolia* in the forage feed base in the humid tropics such as the Amazon piedmont can be used as a tool to both mitigate enteric CH_4_ emissions and to increase animal productivity and hence reduce emissions intensity, and thus reduce pressure on the agricultural frontier in critical areas such as the Amazon.

## Teaser

The inclusion of *T. diversifolia*can be included in low-quality diets of grazing cattle to reduce enteric methane emissions. The use of *T. diversifolia*in grazing systems increases milk production in medium-production cows, and thus it can be an alternative to reduce pressure on the agricultural frontier in critical areas such as the Amazon.

## INTRODUCTION

Methane (CH_4_), despite its relatively short lifetime in the atmosphere (12–15 years), is the second most important greenhouse gas (GHG) of anthropogenic origin with a global warming potential 28 times higher than that of carbon dioxide (CO_2_) (IPCC, 2013). The livestock sector contributes an estimated of 14.5% of global GHG emissions, with CH_4_ from ruminant enteric fermentation accounting for 39.1% of the sector’s emissions and 6% of global emissions (Gerber et al., 2013; Beauchemin et al., 2020).

Extensive cattle ranching systems in tropical and subtropical regions such as the Amazon have caused land degradation, loss of biodiversity, and increased emissions of GHG (Kennedy and Charmley, 2012; Ku-Vera et al., 2020). These systems are also less efficient due to low pasture quality, suboptimal management of resources, hence associated with high carbon footprints (Rao et al., 2015; Carvalho et al., 2020). In the face of current climate problems and the high amounts of GHG generated by livestock systems, major research efforts have focused on reducing enteric CH_4_ emissions through feeding management practices that alter rumen fermentation with the potential to increase animal productivity (Ku-Vera et al., 2020; Valencia-Salazar et al., 2021).

To contribute to sustainable livestock production, these forages must be associated with an increase in milk and meat productivity, with desirable adaptive and nutritional characteristics and a reduction in GHG emissions and other environmental impacts (Tapasco et al., 2019; Arango et al., 2020). Well managed forage base systems, including silvopastoral systems (SPS) could contribute to reduced emissions of enteric CH_4_ (Gaviria-Uribe et al*.,* 2020), nitrous oxide (N_2_O) (Rivera et al., 2018; Rivera and Chará, 2021) and increased carbon accumulation in aboveground biomass and soils (Landholm et al*.,* 2019).

In recent years, tropical trees, and shrubs such as *Tithonia diversifolia* (Hemsl.) A. Gray., when incorporated in SPS have received attention from researchers due to their potential to increase fermentative efficiency and reduce enteric CH_4_ emissions compared to forage species traditionally offered in pastoral diets (Ribeiro et al., 2016; Terry et al., 2016; Galindo-Blanco et al., 2018; Rivera et al., 2021). The benefits of *T. diversifolia* are given by its higher nutritional quality based on high contents of crude protein, minerals, and energy, low fiber values, high degradability, the presence of phytochemical compounds, and its ability to adapt to different edaphoclimatic conditions (Chagas-Paula et al., 2012; Rivera et al., 2021).

Some phytochemical compounds in *T. diversifolia* can decrease enteric CH_4_ production and modify gas production rates due to inhibitory effects on specific groups of rumen microorganisms by their interaction with their membrane or by the interaction with some components of the diet itself (Delgado et al., 2012; Bhatta et al., 2013; Rivera et al., 2021). Delgado et al. (2012) reported that *T. diversifolia* has methane-reducing properties when supplemented at 30% in a feed based on *Cynodon nlemfuensis* and indicated that this was due to the secondary metabolites present in *T. diversifolia*, such as condensed tannins, essential oils, and saponins. Chagas-Paula et al. (2012) reported that *Tithonia* contains over 150 phytochemical compounds, particularly sesquiterpene lactones, diterpenes, flavonoids, tannins, and saponins.

Despite all the above, in vivo experiments with *T. diversifolia* have been limited, especially to determine its CH_4_ mitigation potential in grazing conditions and in an area with extensive land use conflicts such as the Amazon. On the other hand, reducing the high GHG emissions associated with the livestock sector represents an opportunity for countries to move towards achieving their Nationally Determined Contributions (NDCs) under the Paris Agreement (Gaviria-Uribe et al., 2020).

The objective of this study was to evaluate the effects of inclusion of *T. diversifolia* forage to a basal diet based on pasture forage of *B. humidicola* on feed intake, milk yield, and CH_4_ emission from dual-purpose milking cows in the Amazonian piedmont of Colombia.

## MATERIALS AND METHODS

The study was reviewed and approved by Ethics Committee of the Centro Para la Investigación en Sistemas Sostenibles de Producción Agropecuaria (CIPAV) and following protocols of the Colombian law No. 84/1989.

### Location

The study was carried out in El Volga, a commercial dual-purpose cattle farm (N 1°44ʹ34.12″ W 75°15ʹ49.67″) in the Colombian Amazonian piedmont, at an altitude of 347 masl (meters above sea level). Evaluations were made during the moderate rainy season in 2020 (October–November) and the rainy season in 2021 (March–April); according to the historical rainfall regimes of the region, these two moments coincide with the two climatic periods that occur in the study area. The study location is within the regional climate classification defined as tropical rainforest type tropical wet forest—Af (Köppen classification), with a mean annual temperature of 25.5 °C, precipitation of 3800 mm/yr and relative humidity of 85%. The soils are highly weathered and classified as Dystrudepts and Hapludox that originated from fine alluvial sediments (Olaya-Montes et al., 2020).

### Diets and Determination of Their Chemical Composition

The two diets (treatments) evaluated involved two forage species. The basal diet (Diet 1) was fresh cut *Brachiaria humidicola* (Rendle) Schweick harvested from a conventional pasture at a regrowth stage of 30 and 42 days for the moderate rainy and rainy season, respectively. The pasture was a partially degraded grassland, with low presence of tress, and used under extensive grazing. Diet 2 was composed of *T. diversifolia* and *B. humidicola* (15:85, dry matter [DM] basis on average). *B. humidicola* was harvested at the same regrowth stage used for Diet 1. *T. diversifolia* was harvested from a SPS with a density of 2000 plants/ha, associated with trees within the system and along the fences, and the 85:15 ratio was ensured by weighing of the two forages offered to each animal during the measurement days.

**Figure 1.**
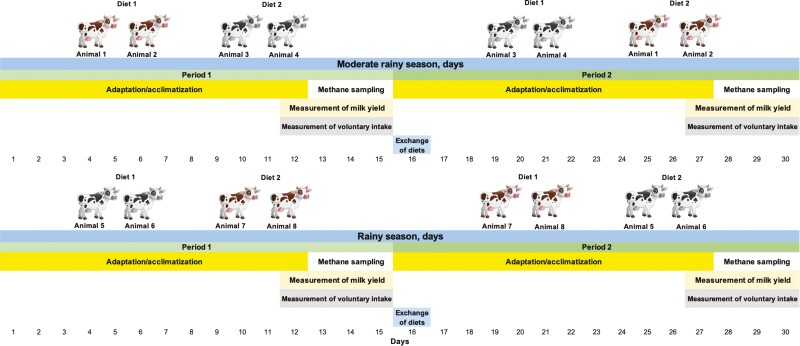
General representation of the experimental design used in the determination of CH_4_ emissions, dry matter intake and milk yield by dual-purpose cows in the moderate rainy and rainy seasons. Diet 1: *B. humidicola* 100%; Diet 2: *T. diversifolia* 15% + *B. humidicola* 85%.

The percentage of shrub inclusion in Diet 2 was estimated during the adaptation period (Figure 1) by measuring pasture biomass in paddocks containing *T. diversifolia* before and after animal grazing four times for each season, using the double sampling technique (Haydock and Shaw, 1975); the proportion of forage species in the diets evaluated was ensured by adjusting the grazing area during the experimental period to guarantee an adequate supply, based on the size and number of animals grazing in the pasture. During the moderate rainy season, the supply of *B. humidicola* in the grazing areas corresponding to Diet 2 was on average 417.5 (± 29.86) kg, and that of *T. diversifolia* was 76.7 (± 7.93) kg/ha of DM. During the rainy season the average supply of *B. humidicola* and *T. diversifolia* was 369.3 (± 27.91) and 64.4 (± 10.68) kg of DM/ha respectively. With this forage supply, a ratio of 84.5:15.5 and 85.2:14.8 was calculated for the moderate rainy and rainy seasons respectively (the individual values are presented in the Supplementary material).

Four samples of each diet were collected during each season to be analyzed in the Animal Nutrition and Forage Quality Laboratory of Centro Internacional de Agricultura Tropical (CIAT, Colombia) (certified by the FAO-IAG proficiency test of feed). Both diets were analyzed for dry matter (DM), crude protein (CP), neutral detergent fiber (NDF), acid detergent fiber (ADF), gross energy (GE), ash (Ash), ethereal extract (EE) and in vitro dry matter degradability (IVDMD). DM content was determined in a forced air oven at 105 °C until constant weight was reached (International Organization for Standardization—ISO 6496) (ISO, 1999), the percentage of N and CP was determined by the Kjeldahl method according to Norma Técnica Colombiana (ICONTEC 4657, 1999), NDF and ADF were determined by the sequential technique described by Van Soest et al. (1991) according to the Association of Official Analytical Chemists (AOAC) 2002.04 and 973.18, respectively (AOAC, 2005a, b, c), and EE by Soxhelet extraction by immersion (Norma Técnica Colombiana—ICONTEC 668, 1973). Ash content was obtained by direct combustion in a muffle furnace at 500 °C according to AOAC 942.05 (AOAC, 2005a, b, c), P (Phosphorus) and Ca (Calcium) by spectrometry, and GE by calorimetry based on ISO 9831 (ISO, 1998) and IVDMD according to Tilley and Terry (1963).

### Animals Evaluated

Eight lactating cows typical of the area with various degrees of crossbreeding (*Bos taurus* × *Bos indicus*) were chosen. For the moderate rainy season, the animals had 232 ± 8.20 days in milk, an age of 64.5 ± 20.4 months, 2.25 ± 1.26 parturitions, live weight of 419 ± 30.6 kg, and produced an average of 5.10 ± 0.59 L/animal/d. For the rainy season, the animals had 186 ± 26.1 days in milk, an age of 80.4 ± 20.9 months, 3.75 ± 2.92 parturitions, live weight of 395 ± 35.1 kg, and milk production of 5.22 ± 1.93 L/animal/d. The animals selected were randomly allocated to the treatments in each season. Mechanical milking was used once a day, milking time was estimated between 7 and 10 min for each animal, and the cows were with the calf during milking. This milking routine was used throughout the experimental period and with the same method and duration for all the animals.

### Milk Production and Compositional Quality

In the last 3 days of the adaptation period and at each gas sampling time (days 12, 13, 14, 15, 27, 28, 29, and 30 of each season—Figure 1), milk production was measured individually, and milk protein and fat production were determined using a LACTOSCAN analyzer. Milk production was corrected for fat and protein content (FPCM—milk standardized at 3.7% and 3.3% fat and protein, respectively) (Thomassen and de Boer, 2005) in order to compare it between diets. During the milk production measurement days, calves were with the cows only to stimulate milking but were separated immediately afterwards to measure real milk production for each diet.

### Estimation of Forage Intake

Diets were offered individually to each animal in feeders installed inside the tunnels during the experimental period. Forages were cut directly from the grazing systems and were offered fresh without chopping (leaves and stems with diameters of less than 5 mm); for Diet 2*, B. humidicola* and *T. diversifolia* were offered separately. All animals had ad libitum access to forages, ensuring the supply ratio of 85:15 of *B. humidicola* and *T. diversifolia* for Diet 2, and water in each compartment. During the adaptation periods the animals were allowed to graze in paddocks with the forages corresponding to their diet with the objective of achieving their normal intake and only entered the polytunnels 6 h/d for adaptation/acclimatization, where the forages of both diets were also offered. No additional concentrates or supplements were added. The voluntary daily intake of each animal, for each of the diets in both seasons was measured four times and was calculated as the difference between the amount of forage offered and rejected. Forages were cut twice per day and offered every three hours while the animals were enclosed in the polytunnels to provide a constant supply of pasture.

### Determination of In Vivo CH_4_ Emissions

Measurements of CH_4_ emissions were performed using the polytunnel technique (Lockyer, 1997; Murray et al., 2007), the study had two 30-d periods (one period in rainy season and one in moderate rainy season). Each season had two 12-d periods of adaptation/acclimatization to the diets and polytunnels, and two 2-d measurement periods with an intermediate day of rest between each sampling day (Figure 1). Gas samples were taken every 60 min starting at 7:00, during 24 h, in 8 mL vials and following the recommendations of Molina et al. (2016) and Gaviria-Uribe et al. (2020); the gas samples were taken using individual extractors at an extraction rate of 0.9 m^3^/s installed in each compartment of the polytunnel, and an environmental sample was also taken every hour (input gas) in order to correct the exhaust gas samples. Every hour, after taking the gas sample, the polytunnel was opened to release the accumulated gas before starting a new measurement. According to Lockyer and Jarvis (1995) and Lockyer (1997), polyethylene structures such as those used in this study have gas recoveries of 95.5–97.9%. Figure 1 describes the general scheme of the experiment.

Total methane emissions were calculated with the ideal gas law according to López and Newbold (2007), based on the concentration of CH_4_ determined by chromatography (ppm) every hour, the total volume of the polytunnel, temperature and atmospheric pressure. The accumulated methane per day (g CH_4_/animal/d) was estimated as the sum of methane production in each hour of the day. Two polytunnels divided into two compartments of 36 m^3^ each were used to house the animals individually. The environmental conditions inside and outside the polytunnels were continuously monitored during the experimental period to ensure that the temperature and humidity inside the structures did not generate thermal stress in the animals and thus ensure normal forage intake.

Methane concentration was measured using a gas chromatograph (Shimadzu GC-2014, SHIMADZU, Japan) with the following specifications: Column: Shimadzu: 1/800 packed stainless steel columns, HayeSep T 80/100 mesh, 4 m HayeSep D 80/100, 1.5 P-N, 0.7 m Shimalite Q 100/180, column temperature: 80 °C, detector temperature: FID = 250 °C, Electron Capture Detector (ECD) 325 °C, methanizer temperature 380 °C, carrier gas: nitrogen, column flow rate 30. 83 mL/min and injection volume managed by a loop with 2 mL capacity (CIAT).

### Experimental Design and Statistical Analysis

A cross-over design was used, and each diet were assigned to animals during the first experimental period and then exchanged between groups for the second period of each season (day 16) (Figure 1). The individual cow was the experimental unit for each variable to measure. Gas emission measurements in each period were made for 2 days in order to account for variations between days and within animals and to have a larger number of measurements. Animal weight and day were used as a covariate and multiple comparisons were evaluated using Tukey’s HSD (honestly significant difference) test, using the RStudio tool (RStudio Team, 2020).

The variables measured were, g CH_4_/animal/d, g CH_4_/kg DM intake, g CH_4_/kg of degraded DM, kg CO_2_-eq/kg of FPCM, kg CO_2_-eq/kg of protein, kg CO_2_-eq/kg of fat, DM intake per animal per day as percentage of live weight, kg FPCM per animal per day, g protein per animal per day, g fat per animal per day and the energy losses as a percent of GE intake (Ym) for each of the diets offered. Before making the contrasts between the means of the variables, normality, homogeneity of variance and additivity of the data were corroborated.

With the division of cumulative CH_4_ emissions (g CH_4_/animal/d) and animal intake per day, g CH_4_/kg DM intake was estimated; g CH_4_/kg of degraded DM was calculated with the g CH_4_/kg DM intake emissions adjusted for the degradability of each of the diets (Table 1), emissions per kg FPCM were calculated by dividing total emissions (g CH_4_/animal/d) by milk production adjusted for FPCM as well as for emissions per kg of fat andprotein, and finally Ym was calculated from the energy contained in each diet, g CH_4_/kg DM intake and the mass energy of CH_4_ (13.3006839 kcal/kg).

**Table 1.** Chemical composition of diets offered to dual-purpose cows in the Colombian Amazonian piedmont

Season	DM	CP	NDF	ADF	Ash	Ca	P	EE	IVDMD	GE
	g/kg of DM									Mcal/kg of DM
Diet 1										
Moderate rainy season	205.7 (± 6.8)	118.9^a^ (± 23.0)	684.5 (± 49.6)	370.4 (± 20.3)	87.1 (± 3.2)	2.3 (± 1.2)	1.7^a^ (± 0.65)	21.5^b^ (± 12.6)	603.1^a^ (± 21.4)	4394.1^a^ (± 57.1)
Rainy season	200.9 (± 8.3)	86.4^b^ (± 2.8)	700.5 (± 73.2)	369.4 (± 40.5)	83.2 (± 02.88)	1.94 (± 0.65)	1.15^b^ (± 0.22)	37.1^a^ (± 2.91)	557.6^b^ (± 59.7)	4217.1^b^ (± 115.7)
*P*-value	0.394	0.046*	0.681	0.965	0.117	0.561	0.001*	0.018*	0.045*	0.043*
SEM	2.62	11.2	17.3	10.5	1.24	0.35	0.14	7.84	13.3	47.46
*T. diversifolia*										
Moderate rainy season	157.1^a^ (± 4.13)	265.1 (± 18.7)	446.1 (± 86.5)	376.3 (± 88.6)	171 (± 30.6)	16.4 (± 1.98)	3.92 (± 1.92)	62.6 (± 2.23)	703.8 (± 2.21)	4327.68^a^ (± 150.6)
Rainy season	139.1^b^ (± 6.4)	264.5 (± 3.6)	484.9 (± 21.6)	383.5 (± 61.6)	162.1 (± 23.2)	19.1 (± 7.21)	3.14 (± 0.61)	53.1 (± 7.02)	696.8 (± 15.9)	4056.8^b^ (± 124.1)
*P*-value	0.003*	0.971	0.418	0.899	0.658	0.502	0.447	0.445	0.625	0.032*
SEM	3.82	7.12	21.9	25.11	9.13	1.84	0.55	5.73	6.42	68.25
Diet 2										
Moderate rainy season	197.4 (± 5.84)	152.1^a^ (± 2.21)	644 (± 6.34)	371.4 (± 5.78)	101.4 (± 2.56)	4.72 (± 1.32)	2.13^a^ (± 0.67)	28.5 (± 2.13)	620.2 (± 2.15)	4382.81^a^ (± 123.5)
Rainy season	190.4 (± 7.8)	116.7^b^ (± 17.1)	663.8 (±5 4.1)	371.8 (± 59.1)	96.6 (± 19.6)	4.85 (± 4.24)	1.44^b^ (± 0.62)	56.4 (± 4.83)	597.9 (± 30.5)	4189.85^b^ (± 120.2)
*P*-value	0.173	0.044*	0.579	0.987	0.127	0.877	0.033*	0.048	0.051	0.040*
SEM	2.49	9.28	16.11	10.33	1.55	0.43	0.16	6.86	8.91	50.2

Diet 1, *B. humidicola* 100%; Diet 2, *T. diversifolia* 15% + *B. humidicola* 85%; DM, dry matter; CP, crude protein; NDF, neutral detergent fiber; ADF, acid detergent fiber; Ash, ash; Ca, calcium; P, phosphorus; EE, ethereal extract; IVDMD, in vitro DM degraded; EB, gross energy.

*Values with different letters in the same column denote significant differences (*P* < 0.05).

## RESULTS

### Chemical Composition of Diets

The chemical composition of the diets offered in both seasons is shown in Table 1. The inclusion of *T. diversifolia* in the basal diet of *B. humidicola* improved CP supply by 17.9 and 35.1% for the moderate rainy and rainy seasons, respectively, and increased IVDMD by 3.17% on average (*P* < 0.05). On the other hand, the supply of this shrub decreased the NDF content by 5.58% with respect to the *B. humidicola* diet and increased mineral content such as Ca and P (*P* < 0.05).

Particularly for *T. diversifolia*, the season influenced DM and GE variables (*P* < 0.05) but not on the other characteristics. On the other hand, in *B. humidicola*, the variables of CP, IVDMD, GE, EE, and P had differences between seasons (*P* < 0.05).

### Forage and Nutrient Intake

Total DM intake expressed as a percentage of animal live weight did not differ between diets (1.84 vs. 1.88 for Diet 1 and Diet 2, respectively) (*P* = 0.369), although on average consumption was higher for Diet 2; on average DM intakes were 1.90 and 1.82% in the rainy and moderate rainy seasons, respectively (*P* = 0.031). For diet 2, the average consumption of *T. diversifolia* was 17.5% (± 2.46) with no differences between seasons (*P* = 0.390) (Table 2). The standard deviation in the supply of *T. diversifolia* during the adaptation/acclimatization period was ± 1.31% and had an average coefficient of variation of 8.4.

**Table 2.** Dry matter (DM) and nutrient intake of dual-purpose cows during the two evaluation seasons in the Colombian Amazonian piedmont

	DM	Ash	NDF	CP	EE	Ca	P	GE
	g/animal/d							Mcal/animal/d
Moderate rainy season								
Diet 1	7,371.3	667.7^b^	5,252.9^a^	855.2^b^	300.2	17.45^b^	12.82^b^	33,310.4
Diet 2	7,651.3	776.2^a^	4,928.9^b^	1,163.1^a^	342.9	35.95^a^	15.72^a^	33,734.2
*P*-value	0.963	0.019*	0.029*	0.003*	0.212	0.003*	0.034*	0.927
SEM	206.9	24.25	148.9	56.75	16.75	3.05	0.71	908.1
Rainy season								
Diet 1	7,412.5	616.9^b^	5,352.1^a^	654.1^b^	348.6^b^	14.26^b^	8.22^b^	31,245.9
Diet 2	7,700.2	756.5^a^	5,040.9^b^	906.4^a^	436.1^a^	39.51^a^	11.61^a^	32,303.2
*P*-value	0.557	0.026*	0.028*	0.013	0.012*	<0.001*	0.014*	0.621
SEM	242.7	32.48	125.4	53.6	18.48	4.23	0.74	1022.6

Diet 1, *B. humidicola* 100%; Diet 2, *T. diversifolia* 15% + *B. humidicola* 85%; DM, dry matter; CP, crude protein; NDF, neutral detergent fiber; Ca, calcium; P, phosphorus; EE, ethereal extract; GE, gross energy; SEM, standard error of the mean.

*Values with different letters in the same column denote significant differences (*P* < 0.05).

### Milk Production and Quality

**Figure 2.**
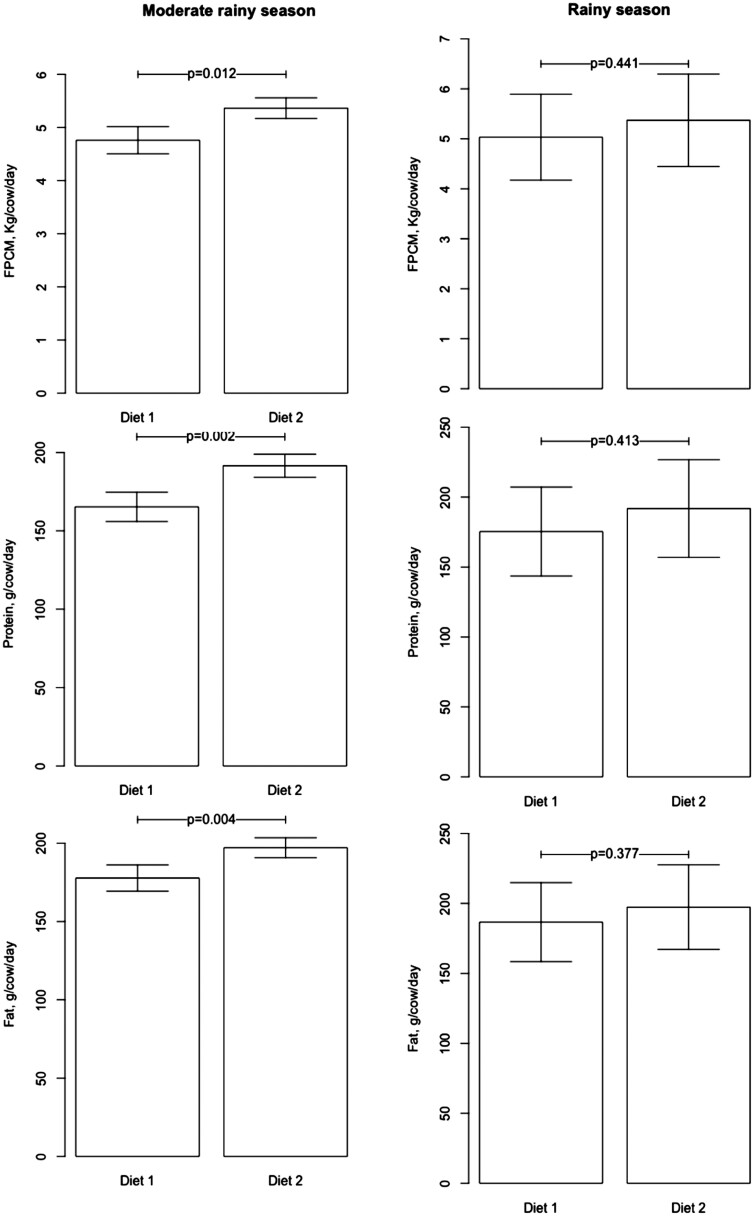
Milk yield and solids produced by dual-purpose cows with the two diets evaluated in the Amazonian Piedmont of Colombia. FPCM: Fat and protein corrected milk; Diet 1: *B. humidicola* 100%; Diet 2: *T. diversifolia* 15% + *B. humidicola* 85%.

FPCM production during the moderate rainy season was 4.81 (± 0.50) and 5.36 (± 0.36) and in the rainy season was 5.02 (± 1.62) and 5.37 (± 1.74) kg/animal/d for Diet 1 and Diet 2, respectively. In the moderate rainy season, there were significant differences between diets for FPCM, g of fat per animal per day and g of protein per animal per day (*P* < 0.05), but in the rainy season there were no differences (*P* > 0.05) although Diet 2 had higher productivity (Figure 2).

### Methane Yield

**Figure 3.**
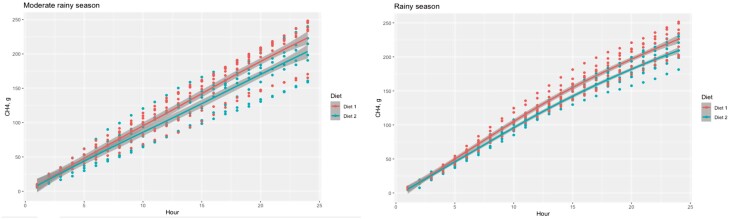
Mean cumulative methane production of dual-purpose cows (g/animal/d) during the two evaluation seasons (shaded lines represent SEM at each time). Diet 1: *B. humidicola* 100%; Diet 2: *T. diversifolia* 15% + *B. humidicola* 85%.

Figure 3 shows the cumulative CH_4_ production by enteric fermentation (g/animal/d) during the two seasons. The *B. humidicola* + *T. diversifolia* diet generated lower emissions in each of the variables measured (*P* < 0.05). Table 3 shows the results related to CH_4_ yield.

**Table 3.** Methane yield in dual-purpose cows fed with *B. humidicola* and *B. humidicola* + *T. diversifolia* diets in the Amazon piedmont of Colombia

	g of CH_4_/animal/d	g of CH_4_/kg of DMI	g of CH_4_/kg of DMD	kg of CO_2_/kg of FPCM**	kg of CO_2_/kg of protein**	kg of CO_2_/kg of fat**	Ym (%)
Moderate rainy season							
Diet 1	218.3 (± 27.6)^a^	27.58 (± 1.13)^a^	45.39 (± 1.92)^b^	1.41 (± 0.17)^a^	39.65 (± 4.78)^a^	40.03 (± 4.83)^a^	8.20 (± 0.59)^a^
Diet 2	207.6 (± 35.2)^b^	25.45 (± 1.06)^b^	41.11 (± 3.04)^a^	1.19 (± 0.17)^b^	32.43 (± 4.88)^b^	32.36 (± 4.87)^b^	7.58 (± 0.38)^b^
*P*-value	0.003*	0.004*	0.002*	0.002*	<0.001*	<0.001*	<0.001*
SEM	8.39	0.29	0.84	0.05	1.59	1.63	0.32
Rainy season							
Diet 1	228.1 (± 33.8)^a^	28.37 (± 1.13)^a^	50.70 (± 3.18)^b^	1.38 (± 0.5)^a^	40.1 (± 5.37)^a^	36.6 (± 6.02)^a^	8.87 (± 0.59)^a^
Diet 2	208.5 (± 35.15)^b^	26.19 (± 1.06)^b^	43.87 (± 5.02)^a^	1.11 (± 0.31)^b^	33.1 (± 4.87)^b^	30.3 (± 4.47)^b^	8.07 (± 0.38)^b^
*P*-value	0.016*	0.006*	<0.001*	0.011*	0.042*	0.028*	0.006*
SEM	5.11	0.41	1.34	0.05	1.41	1.46	0.54

Diet 1, *B. humidicola* 100%; Diet 2, *T. diversifolia* 15% + *B. humidicola* 85%; DMI, dry matter intake; DMD, degraded DM; FPCM, fat and protein corrected milk (kg); Ym, percentage of GE emitted as CH_4_; SEM, standard error of the mean.

*Values with different letters in the same column denote significant difference (*P* < 0.05).

**CO_2_ equivalent based on methane from enteric fermentation (The Global Warming Potential (GWP) of the pollutants over a time-horizon of 100 years (were: 28 for CH_4_; 265 for N_2_O).

In both seasons, *T. diversifolia* intake had a significant effect on emissions and emission intensity for FPCM, kg of protein and kg of fat. Energy loss in the form of methane (Ym) was greater in the *B. humidicola*-based diets (Diet 1) (*P* < 0.05) (Table 3).

Finally, regarding environmental conditions, average temperature in the moderate rainy season was 29.0 (± 2.4) and 27.1 (± 8.2)°C, and relative humidity was 83.7 (± 6.1) and 78.6 (± 8.2)%, inside and outside the polytunnels, respectively. For the rainy season period, temperature was 27.8 (± 2.9) and 26.1 (± 10.2)°C, and humidity was 87.1 (± 4.0) and 83.9 (± 9.5)%, inside and outside the polytunnels, respectively. The maximum temperature inside the polytunnels was 34.0 °C (between 14:00 and 16:00 h) and the maximum humidity was 90% (between 4:00 and 6:00 h).

## DISCUSSION

### Chemical Composition of Diets and Nutrient Intake

The results of this study show that although *T. diversifolia* is not a leguminous species, its CP values are as high as those reported in some tropical legumes such as *Stylosanthes guianensis* (Morgado et al., 2009), *Arachis pintoi* (Khan et al., 2013), *Leucaena diversifolia* and *L. leucocephala* (Gaviria-Uribe et al., 2020), and *Gliricidia sepium* (Silva et al., 2017), and higher than those observed in most tropical grasses (Carvalho et al., 2017; Ribeiro et al., 2017; Rivera et al., 2021). Likewise, NDF and ADF content of *T. diversifolia* is lower than the common values observed for tropical forages (Souza et al., 2007), a property that favors adequate voluntary intake and improves nutrient degradability. *T. diversifolia* had two and three times more CP, 30 and 34% less NDF and 16 and 20% more IVDMD than *B. humidicola*, so the mixture that included only 17.5% of *T. diversifolia* favored a greater supply and intake of nutrients, especially CP and minerals, and a reduction in the amount of NDF compared to the *B. humidicola* base diet.

However, although the results showed an improvement in nutritional quality in the diets with *T. diversifolia*, no significant increase in total DM intake was found, which could be due to the moderate inclusion of the shrub in the diet and to the relatively low productive level of the cows under evaluation. Other studies have found an increase in DM intake when the shrub *Leucaena leucocephala* replaced 15–25% of a grass-based diet (Cuartas et al., 2015; Molina et al., 2016); this condition could favor higher emissions per animal day due to higher DM intake, but in this study this behavior did not occur.

The DM intake as a percentage of live weight found in this study coincides with values reported in other studies under tropical conditions. According to Boval et al. (2015), Piñeiro-Vázquez et al. (2017) and Gaviria-Uribe et al. (2020), under tropical conditions DM intake for grazing cattle ranges between 1.5 and 2.1% of animal live weight; in addition, estimates from models such as Cornell Net Carbohydrate and Protein System (CNCPS) (Tylutki et al., 2008) corroborate these relatively low values, since nutritional requirements are not high, and diets have low passage rates and high fiber contents.

Among the evaluation seasons, differences in CP, P, GE, and IVDMD were identified for *B. humidicola*, which is probably due to the post-grazing pasture recovery times (30 vs. 42 d for moderate rainy and rainy seasons, respectively), as the rainy season in the study area delays pasture recovery due to high soil moisture. Longer resting times can generate higher NDF contents and lower CP and IVDMD values due to higher tissue cell wall production that causes nutrient translocation in the plant (Dias et al., 2019). The differences in the *B. humidicola + T. diversifolia* diet were probably due to modifications in the chemical composition of *B. humidicola* which was the species that contributed the highest amount of total DM in the diet because *T. diversifolia* did not vary considerably between seasons. Rivera et al. (2021) have found that the nutritional quality of *T. diversifolia* does not vary considerably with regrowth time.

Regarding *T. diversifolia* intake as a percentage of the diet, the values of this study are higher than those reported by Ribeiro et al. (2016) and Mejía-Díaz et al. (2017) who found consumption of 5–15% of the total DM in the diet and below those found by Gallego-Castro et al. (2017) who obtained consumptions of up to 25% of the total DM, with productive and economic benefits in high production cows (production > 20 L/animal/d). These intake values confirm the possibility of achieving significant inclusions of *T. diversifolia* in bovine systems with the objective of increasing animal production and overall system efficiency. It has been shown that the effects of *T. diversifolia* could occur when this shrub represents more than 15% of DM of the total diet (Delgado et al., 2012; Rivera et al., 2021). In the present study, with a consumption of *T. diversifolia* equivalent to 17.5% of the total DMI, the animals received 19% more minerals, 37% more CP, 30% more P and 2.4 times more Ca per day, nutrients that are very important in dairy production. It also represented a reduction in the ADF intake that was significantly lower than the amount consumed by cows without the inclusion of *T. diversifolia*. Since these animals have medium nutritional requirements given their milk production, and are in the second third of lactation, the increase in nutrients offered in Diet 2 could have a greater effect on the animals.

### Milk Production and Compositional Quality

Despite the lack of differences in the DM intake between treatments, the inclusion of *T. diversifolia* in the diet significantly increased production of milk, fat, and protein per day (with increased nutrient consumption), this condition could represent a potential for not having to expand the livestock frontier, given that greater production is being obtained with the same amount of area, but with a higher quality of DM consumed. As previously presented, this species increases CP, fat, and mineral contents of the diet, improves DM degradability, and decreases fiber contents (NDF and FDA) with improved milk production and quality. In addition, authors such as La O et al. (2012), Gallego-Castro et al. (2017), Rivera et al. (2021) and Valencia-Salazar et al. (2021) have found that *T. diversifolia* modifies the fermentation dynamics of diets containing it compared to grass-only diets (a shorter lag phase and a higher rapidly degradable fraction that enhance a better rumen nutrient balance and a higher availability of nutrients in the rumen).

Similar results have been found by other authors. Ribeiro et al. (2016) evaluated the effects of replacing fresh sugarcane and concentrates with *T. diversifolia* (0, 6, 4, 15%) in lactating cows, reporting that it was possible to replace sugarcane (20% of DM) and concentrate (11.2% of DM) without any change in total intake (18.7 kg DM/d), milk production (22.9 kg/d) and nutritional composition. Similarly, the inclusion of *T. diversifolia* did not negatively affect glucose, urea, triglycerides, cholesterol, non-esterified fatty acids and ß-hydroxybutyrate parameters. On the other hand, in Colombia, Rivera et al. (2015), when comparing a monoculture system of *B. brizantha* and a system with *T. diversifolia* (3500 shrubs/ha), found that the latter increased milk production (kg per cow per day or kg/ha/d), non-fat solids (kg per cow per day or kg/ha/d) and total solids (kg per cow per day or kg/ha/d). Daily milk production per cow with *T. diversifolia* was 4.92 kg, 7% more than the system with *B. brizantha*. Also, the animal stocking rate increased 32%, producer income increased by 25% and benefits for the dairy industry were achieved, since there was a greater volume of milk per hectare (29% more) with higher solids content (*P* < 0.05) and less seasonality in production.

The higher milk solids yield with *T. diversifolia* inclusion during the moderate rainy season was probably due to a better energy-protein balance and greater solubility at the rumen level during early fermentation times; this favors a greater availability of nutrients, a greater production of microorganisms and a better synchrony between energy and protein in the rumen. According to Gallego-Castro et al. (2017), *T. diversifolia* provides higher nonstructural carbohydrates (11.2%) than tropical pastures and its protein is rapidly soluble (> 40%). These characteristics have also been reported by La O et al. (2012) who found that diets with *T. diversifolia* have a higher degradation fraction than that reported for diets based only on grasses, improving fermentation dynamics, which favors a greater and faster availability of nutrients in the rumen.

Finally, the milk production found in this study is similar to that reported by Rivera et al. (2015) and Parra et al. (2017), who found average productions ranging from 2 to 6 L/animal/d in dual-purpose cows in this region.

### CH_4_ Emissions

The inclusion of *T. diversifolia* significantly contributed to reduce the emissions of CH_4_ per day and per unit of DM consumed or degraded. The g of CH_4_/kg DM consumed and Ym are slightly above those reported by Rivera et al. (2021), who evaluated different genotypes of *T. diversifolia* under in vitro conditions in a 25:75 mixture of *T. diversifolia* and *B. brizantha* and found values between 24.1 to 26.4 and 7.80 to 8.76 for g CH_4_/kg DM consumed and Ym, respectively. These values are also close to those reported by the IPCC guidelines (Gavrilova et al., 2019) for pastoral diets in tropical environments (6.5%) but could be used to estimate GHG inventories more in line with the conditions present in the study area, since the results were obtained at two times of the year and under the usual conditions of the production systems in the region.

Ribeiro et al. (2016) evaluated the substitution (up to 15%) of concentrate feed by this shrub in high milk production cows, and although metabolic and productive parameters were not affected, CH_4_ emissions increased when *T. diversifolia* was included since the base diet was of high quality. Therefore, the results presented in this research provide important new knowledge about an additional species with the ability to mitigate CH_4_ emissions by enteric fermentation in tropical and subtropical conditions with medium to low-quality forage, since *T. diversifolia* can be used from sea level to 2200 masl and from soils with moderate fertility to acid soils with low organic matter and Al saturation with limited cation exchange capacity (Ruiz et al., 2013; Holguín et al., 2015).

The mechanisms by which *T. diversifolia* mitigates CH_4_ emissions may be diverse. The decrease in fiber, the increase in CP and digestibility and the contribution of some phytochemical compounds are among the possible mechanisms when the inclusion in the diet is representative (> 15% of the total DM intake). The inclusion of *T. diversifolia* in ruminant diets has been proposed as a mitigation alternative due to its high degradability and low fiber content, as these characteristics have been associated with lower CH_4_ emissions by enteric fermentation (Yan et al., 2006; Gaviria-Uribe et al., 2020; Valencia-Salazar et al., 2021). An additional factor that can contribute to reduce enteric emissions is the presence of phytochemical compounds such as sesquiterpene lactones, diterpenes, flavonoids, tannins, and saponins that can modify the population of methanogenic microorganisms in the rumen due to the interaction with their membrane or with some components of the diet itself (Chagas-Paula et al., 2012; Delgado et al., 2012; Rivera et al., 2021).

Low contents of ADF (<40%) and NDF (<50%), acceptable amounts of soluble carbohydrates (>12%), high degradability (>70%) and high contents of CP (>20%) appear to be the main proximal features that decrease CH_4_ at the ruminal level (Rivera et al., 2021). Yan et al. (2006) reported that reducing the contents of NDF and ADF to 1% reduced the CH_4_ emissions per kg of IDM by 2.01 and 2.26 l, respectively. These authors also reported that for every 1% increase in protein content, emission of enteric CH_4_ decreased by 6.22 L/kg of DM consumed. Similarly, the consumption of less lignified grasses has a clear effect on ruminal digestibility and passage rate (O` Mara, 2004). Thus, Blaxter and Clapperton (1965) reported that by decreasing the digestibility of forages from 75 to 55%, the emission of methane increases from 306 to 499 g/d. Lower fiber content and higher CP content could explain the CH_4_ decrease found in this study by the inclusion of *T. diversifolia* in a basal diet of *B. brizantha* or another tropical pasture.

**Figure 4.**
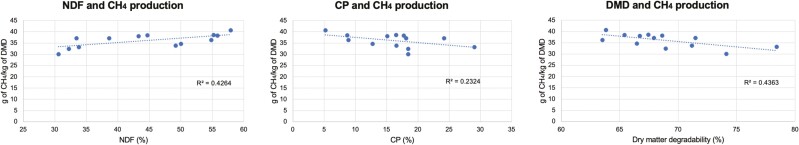
Relationship between some chemical characteristics and CH_4_ emissions in different studies with the inclusion of *T. diversifolia.* Charts elaborated from the studies of: Galindo et al. (2014), Ribeiro et al. (2016), Valentina-Salazar et al. (2021), Holguín-Castaño et al. (2021), Huertas et al. (2021) and Rivera et al. (2021).

For example, Figure 4 shows the relationship between NDF and CP content, and the DM degradability with CH_4_ generation in different studies with *T. diversifolia*, finding a relationship between these chemical characteristics in the feed and CH_4_ emissions; high values of NDF are associated with higher CH_4_ generation, and high contents of CP and DM degradability are associated with lower emissions.

On the other hand, even though the main phytochemical compounds in this study were not determined, authors such as Delgado et al. (2012) and Valencia-Salazar et al. (2021) have identified that *T. diversifolia* can provide acceptable values of alkaloids, flavonoids, and saponins, phytochemical compounds with potential to modify some methanogenic populations in the rumen. On the other hand, Rivera et al. (2021) reported mean values for tannins in this species but have highlighted that it is important to characterize these secondary metabolites since their mitigation potential will depend on their structure (Barahona et al. 2006).

Some studies have evaluated the effect of *T. diversifolia* on CH_4_ emissions (Ribeiro et al., 2016; Terry et al., 2016; Rivera et al., 2021), although the results are divergent. According to the results of these studies, the effect of *T. diversifolia* on CH_4_ production depends on the percentage of inclusion in the diet, the quality of base diet and apparently some ecotypes of this species have different effects on CH_4_ emissions (Rivera et al., 2021). Rivera et al. (2021) found that *T. diversifolia* favors the production of propionic and butyric acid instead of acetic acid and decreases the lag phase of gas production, in addition to improving DM degradability and thus nutrient availability in *B. brizantha* basal diets, which is associated with lower CH_4_ production in the rumen due to the generation of lower amounts of free hydrogens that can be used in other metabolic pathways.

Galindo-Blanco et al. (2018) demonstrated that the inclusion of *T. diversifolia* can reduce the population of rumen protozoa and methanogenic microorganisms when evaluated under in vitro conditions at levels above 15% of the total DM, and according to Delgado et al. (2012) the addition of 30% *T. diversifolia* to a diet based on *Cynodon nlemfuensis* generated 32.9% less CH_4_ production in in vitro studies, probably due to the presence of secondary metabolites of *T. diversifolia*, such as tannins, flavonoids, saponins, and alkaloids that reduce rumen protozoan populations, which share a symbiotic relationship with ruminal CH_4_-producing methanogens (Hook et al., 2010).

Finally, regarding the mitigation per unit of product achieved in the *B. humidicola* + *T. diversifolia* diet, it is highlighted that this was due to both the reduction of total CH_4_ (CH_4_/animal/d) and the productive increase especially in the moderate rainy season. According to Rivera et al. (2016) and Gonzáles-Quintero et al. (2020), an alternative to decrease emission intensities is oriented to improve productive efficiency, that is, to increase production. This not only improves the environmental condition of the systems but can also improve their economics.

It is recommended to increase knowledge about the phytochemical components of *T. diversifolia* that may affect CH_4_ emissions, in addition to more holistic research such as Life cycle assessment (LCA) and with gas balance including the carbon sequestration potential of such systems.

## CONCLUSIONS

The results of this study demonstrate that under low and humid tropical conditions, the inclusion of a relatively low amount of *T. diversifolia* in the diet of dairy cows contribute to the mitigation enteric CH_4_ emissions on in vivo conditions in different climatic periods of the year, reduce emission intensities, and improve milk production and compositional quality. This is particularly relevant for tropical countries, where breeding and dual-purpose cows are the groups with highest contribution to CH_4_ emissions and this reduction could be achieved with a resource produced in the farm at a relatively low cost. Currently, in countries such as Colombia, the information about emission factors in local conditions is scarce. Therefore, the emission factors determined in this study could contribute to improve regional GHG inventories, and the promising mitigation results could provide alternatives to achieve the NDCs of countries such as Colombia and offer options to improve livestock systems and reduce pressures on the agricultural frontier in critical areas of the Amazon.

## Supplementary Material

txac139_suppl_Supplementary_File

## Data Availability

The raw data supporting the conclusions of this article will be made available by the authors, without undue reservation.

## Abbreviations

ADF: acid detergent fiber
Af: Tropical wet climate
AOAC: Association of Official Analytical Chemists
Ca: Calcium
CH_4_: methane
CIAT: *Centro Internacional de Agricultura Tropical*
CIPAV: *Centro Para la Investigación en Sistemas Sostenibles de Producción Agropecuaria*
CO_2_: carbon dioxide
CP: crude protein
DM: dry matter
DMD: dry matter degraded
DMI: dry matter intake
ECD: Electron Capture Detector
EE: ethereal extract
FAO-IAG: The Food and Agriculture Organization—Industry Advisory Group
Fig: Figure
FPCM: fat and protein corrected milk (kg)
GE: gross energy
GHG: greenhouse gas
GWP: Global Warming Potential
ha: hectare
HSD: honestly significant difference
IPCC: The Intergovernmental Panel on Climate Change
ISO: International Organization for Standardization
IVDMD: in vitro dry matter degradability
kcal: kilocalories
kg: kilogram
l: liters
LCA: Life cycle assessment
m^3^: Cubic meter;
masl: meters above sea level
Mcal: Megacalorie
mL: milliliter; mm, millimeters;
N: north
N_2_O: nitrous oxide
NDCs: Nationally Determined Contributions
NDF: neutral detergent fiber
NTC: Norma técnica Colombiana
P: Phosphorus
ppm: parts per million
SPS: silvopastoral systems
W: west
Ym: energy losses as a percent of GE intake
